# Incidence and Risk Factors for Permanent Pacemaker Implantation After Tricuspid Valve Repair

**DOI:** 10.1016/j.atssr.2024.08.003

**Published:** 2024-08-28

**Authors:** Jae Woong Choi, Muath Bishawi, Carmelo Milano, Jeffrey Gaca, Keith Carr, Andrew Wang, Donald D. Glower

**Affiliations:** 1 Division of Cardiothoracic Surgery, Department of Surgery, Duke University Medical Center, Durham, North Carolina; 2 Division of Cardiology, Department of Medicine, Duke University Medical Center, Durham, North Carolina; 3 Department of Thoracic and Cardiovascular Surgery, Seoul National University Hospital, Seoul National University College of Medicine, Seoul, Republic of Korea

## Abstract

**Background:**

Although the likelihood of needing a permanent pacemaker (PPM) after tricuspid valve (TV) repair has been thought to be low compared with TV replacement, the incidence and determinants are controversial. This study aimed to evaluate the incidence and risk factors for PPM implantation after TV repair.

**Methods:**

A total of 1237 consecutive patients undergoing TV repair from 1997 to 2019 were reviewed using a prospectively maintained database, and 1058 patients were enrolled.

**Results:**

Incidence of PPM implantation was 10.3% (n = 109). Median time to PPM implantation was 7 (range, 6-9) days. Indications for PPM implantation were heart block (n = 62, 56.9%), junctional or sinus bradycardia (n = 21, 19.3%), and ventricular arrhythmia (n = 17, 15.6%). Likelihood of PPM varied with concurrent procedures: left ventricular assist device (3.4%), mitral repair (11.2%), mitral valve replacement (13.7%), aortic and mitral valve replacement (19.2%), and isolated tricuspid repair (6.5%). Older age (odds ratio [OR], 1.020; 95% CI, 1.003-1.036), prior mediastinal radiation (OR, 4.106; 95% CI, 1.598-10.554), and concomitant mitral and aortic valve replacement (OR, 1.963; 95% CI, 1.046-3.683) were risk factors, and concomitant left ventricular assist device implantation (OR, 0.325; 95% CI, 0.139-0.759) was a protective factor for PPM implantation. PPM implantation did not affect the early outcomes, overall survival (*P* =.287), or cumulative incidence of recurrent moderate or greater tricuspid regurgitation (*P* =.890) or TV reoperation (*P* =.602).

**Conclusions:**

The likelihood of PPM implantation after TV repair is relatively high, but PPM implantation does not affect the early and long-term clinical outcomes. Older age, prior mediastinal radiation, and concomitant mitral and aortic valve replacement are risk factors, and concomitant left ventricular assist device implantation is a protective factor for PPM implantation after TV repair.

## Visual Abstract

**Figure:**
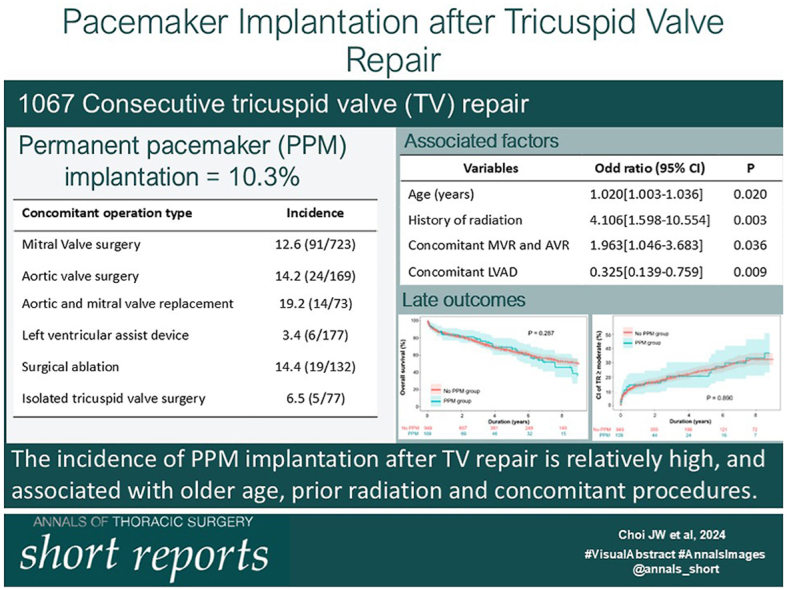

**In Short**

▪ The likelihood of permanent pacemaker implantation after tricuspid repair is relatively high, but permanent pacemaker implantation does not affect the early and long-term outcomes.
▪ Older age, prior mediastinal radiation, and concomitant mitral and aortic valve replacement are risk factors, and concomitant left ventricular assisting device implantation is a protective factor for permanent pacemaker implantation after tricuspid valve repair.

The need for new pacemaker placement has long been known to be increased by tricuspid valve replacement.^1^^,^^2^ However, the likelihood of needing a new pacemaker after tricuspid valve repair has been thought to be low.^3^ Debate about the safety of tricuspid repair led to a recent Cardiothoracic Network trial randomizing mitral repair patients to concurrent tricuspid repair vs control for moderate tricuspid regurgitation (TR) or significant tricuspid annular dilation.^4^ When this trial showed that tricuspid repair was associated with a 14% likelihood of needing a new pacemaker at the time of mitral repair, many questioned the safety of concurrent tricuspid repair for anything less severe than TR. Along those lines, pacemaker insertion after aortic valve replacement has been reported to impair late survival.^5^ Questions were also raised whether the 14% likelihood of needing a new pacemaker was a technical issue vs a synergy between concurrent mitral and tricuspid valve surgery increasing atrioventricular nodal injury.

To settle these issues, this study aimed to evaluate the incidence of permanent pacemaker (PPM) implantation, and risk factors for PPM implantation after tricuspid valve repair.

## Material and Methods

### Methods

After institutional review board approval to waive patient consent (IRBPro00105933, June 22, 2020), a prospectively maintained database was used to identify all consecutive patients undergoing tricuspid valve annuloplasty between 1997 and 2019 (N = 1237). Among them, 1058 patients were enrolled in this study, patients were excluded for preoperative pacemaker, heart or lung transplantation, chronic pulmonary embolism, or endocarditis.

Recurrence of TR was documented by echocardiography using American Society of Echocardiography guidelines. The total number of echocardiogram procedures is 3551, with a median follow-up duration of 0.83 years (range, 0.04-3.43 years). During follow-up, echocardiography was performed at least once in 1013 patients (95.7%), and more than 2 echocardiography was performed in 822 patients (77.7%).

If a patient developed significant bradycardia beyond 5 days, PPM implantation was considered.

### Statistical Analysis

Statistical software was performed using the IBM SPSS statistical software (version 25.0, IBM Inc) and R statistical Software (version 4.3.2., 2023; r-project.org). Continuous variables are presented as mean ± SD or median (interquartile range). The categorical variables are presented as the number and percentage. Survival curves were compared using the log-rank test. Cumulative incidence curves were estimated, considering death, insertion of a left-ventricular assist device (LVAD), or cardiac transplantation, and compared using Gray’s test. Logistic regression analysis was performed to find independent predictors of PPM implantation. Variables with a *P* value <.05 in the univariate analysis were entered into the multivariable model.

## Results

### Preoperative and Operative Characteristics

Of all the patients, 564 (53.4%) patients had atrial fibrillation, and 526 (51.0%) patients had severe TR. The mean left ventricular ejection fraction was 42.9%, and the most common TV disease etiology was functional (928 of 1058, 87.7%). All patients received tricuspid annuloplasty, and 1021 of 1058 (96.5%) annuloplasty patients received a prosthetic annuloplasty ring. Four types ring were primarily used (Supplemental Table 1). Concurrent procedures included mitral valve replacement (MVR) (n = 405, 38%), mitral valve repair (n = 322, 30.2%), aortic valve replacement (AVR) (n = 165, 15.5%), MVR and AVR (n = 74, 6.9%), and LVAD implantation (n = 177, 16.6%).

The preoperative and operative characteristics according to PPM implantation are summarized in Table 1. Patients receiving pacemakers were older and more likely to have received prior mediastinal radiation. Patients receiving pacemakers were more likely to have concurrent MVR, double valve replacement (MVR and AVR), or coronary artery bypass grafting and were less likely to receive a LVAD implantation.

**Table 1:** Preoperative and Operative Characteristics

Variables	All (N = 1058)	PPM (n = 109)	No PPM (n = 949)	*P* Value
Preoperative characteristics				
Age, y	62 ± 14	65 ± 13	61 ± 14	.006
Male	476 (45.0)	49 (45.0)	427 (45.0)	.986
NYHA functional class ≥3	722 (68.3)	68 (62.4)	654 (69.0)	.161
Diabetes	279 (26.4)	26 (23.9)	253 (26.7)	.521
Hypertension	711 (67.3)	75 (68.8)	636 (67.1)	.717
Atrial fibrillation	564 (53.4)	64 (58.7)	500 (52.7)	.236
Chronic renal failure	458 (44.1)	50 (46.3)	408 (43.8)	.624
Severe tricuspid regurgitation	526 (51.0)	47 (43.9)	479 (51.8)	.121
TV etiology, functional	928 (87.7)	101 (92.6)	827 (87.1)	.253
History of cardiac surgery	345 (32.6)	36 (33.0)	309 (32.6)	.922
Coronary artery disease	327 (30.9)	40 (36.7)	287 (30.3)	.169
History of radiation	21 (2.0)	7 (6.4)	14 (1.5)	.003
Left ventricular ejection fraction, %	42.9 ± 15.7	43.9 ± 13.8	42.8 ± 15.9	.401
Operative characteristics				
Tricuspid valve annuloplasty				
DeVega annuloplasty	37 (3.5)	2 (1.8)	35 (3.7)	.419
Prosthetic ring annuloplasty	1021 (96.5)	107 (98.2)	914 (96.3)	.419
CABG	159 (15.0)	25 (22.9)	134 (14.1)	.015
Mitral valve repair	321 (30.3)	36 (33.0)	285 (30.0)	.519
MVR	402 (38.0)	55 (50.5)	347 (36.6)	.005
AVR	161 (15.2)	23 (21.1)	138 (14.5)	.071
MVR and AVR	73 (6.9)	14 (12.8)	59 (6.2)	.010
Surgical ablation	132 (12.5)	19 (17.4)	113 (11.9)	.098
LVAD implantation	177 (16.7)	6 (5.5)	171 (18.0)	<.001
Isolated tricuspid surgery	77 (7.3)	5 (4.6)	72 (7.6)	.254
Cardiopulmonary bypass time, min	216 ± 83	218 ± 71	215 ± 85	.696
Aortic cross clamp time, min	117 ± 54	120 ± 47	116 ± 55	.510

Values are presented as mean ± SD or n (%).

AVR, aortic valve replacement; CABG, coronary artery bypass grafting; LVAD, left ventricular assist device; MVR, mitral valve replacement; NYHA, New York Heart Association; PPM, permanent pacemaker; TV, tricuspid valve.

### PPM Implantation After TV Repair

A total of 109 patients (10.3%) required PPM implantation before discharge. The median time to PPM implantation was 7 (6-9) days. The indication for PPM implantation were heart block (n = 62, 56.9%), junctional or sinus bradycardia (n = 21, 19.3%), ventricular arrhythmia (n = 17, 15.6%), and unknown (n = 9, 8.3%).

The likelihood of pacemaker placement varied with concurrent procedures (Table 2): LVAD (6 of 177, 3.4%), mitral repair (36 of 321, 11.2%), MVR (55 of 402, 13.7%), aortic and mitral replacement (14 of 73, 19.2%), CABG (25 of 159, 15.7%), and isolated tricuspid repair (5 of 77, 6.5%). The pacemaker requirement at the time of mitral repair for degenerative disease (23 of 154, 13.0%) was similar to the incidence of PPM after MVR. Two preoperative conditions and 2 types of concomitant surgery were associated with pacemaker implantation after TV repair in multivariable analyses. Older age (odds ratio [OR], 1.020; 95% CI, 1.003-1.036), prior mediastinal radiation (OR, 4.106; 95% CI, 1.598-10.554), and concomitant MVR and AVR (OR, 1.963; 95% CI, 1.046-3.683) were risk factors, and concomitant LVAD implantation (OR, 0.325; 95% CI, 0.139-0.759) was a protective factor for PPM implantation after TV repair (Table 3).

**Table 2:** Permanent Pacemaker Implantation Incidence by Operation Type

Operation Type	Incidence
All tricuspid repair	10.3 (109/1057)
Concomitant mitral valve surgery	12.6 (91/723)
Mitral repair	11.2 (36/321)
For degenerative mitral regurgitation	13.0 (23/154)
MVR	13.7 (55/402)
Concomitant aortic valve surgery	14.2 (24/169)
AVR	14.3 (23/161)
Aortic valve repair	16.5 (1/8)
Concomitant double valve surgery	17.4 (19/111)
AVR + MVR	19.2 (14/73)
AVR + mitral repair	10.8 (4/37)
Concomitant LVAD	3.4 (6/177)
Concomitant CABG	15.7 (25/159)
Concomitant surgical ablation	14.4 (19/132)
Isolated tricuspid surgery	6.5 (5/77)

Values are presented as % (n/N).

AVR, aortic valve replacement; CABG, coronary artery bypass grafting; LVAD, left ventricular assist device; MVR, mitral valve replacement.

**Table 3:** Multivariable Risk Factor Analysis for Permanent Pacemaker Implantation

Variables	Univariate Analysis		Multivariable Analysis	
	OR (95% CI)	*P* Value	OR (95% CI)	*P* Value
Age	1.023 (1.007-1.039)	.006	1.020 (1.003-1.036)	.020
History of radiation	4.583 (1.808-11.617)	.001	4.106 (1.598-10.554)	.003
Concomitant MVR	1.767 (1.187-2.631)	.005		
Concomitant AVR and MVR	2.223 (1.196-4.132)	.012	1.963 (1.046-3.683)	.036
Concomitant CABG	1.810 (1.117-2.993)	.016		
Concomitant LVAD	0.265 (0.114-0.614)	.002	0.325 (0.139-0.759)	.009

AVR, aortic valve replacement; CABG, coronary artery bypass grafting; LVAD, left ventricular assist device; MVR, mitral valve replacement; OR, odds ratio.

### Early and Long-Term Clinical Outcomes

The overall operative mortality was 6.1%, without difference between patients with PPM and without PPM. The patients requiring PPM implantation showed lower incidence of acute kidney injury, reoperation for bleeding, and longer hospital stay, but there were no statistically significant differences (Supplemental Table 2). In the long-term data, the overall 5-year survival was 65%. The 5-year cumulative incidence of moderate or greater TR or TV reoperation were 24.2% and 1.0%, respectively. PPM implantation did not affect the overall survival(*P* = .287), cumulative incidence of recurrence of moderate or greater TR (*P* = .890), or TV reoperation (*P* = .602) (Supplemental Table 2; Figure)

**Figure:**
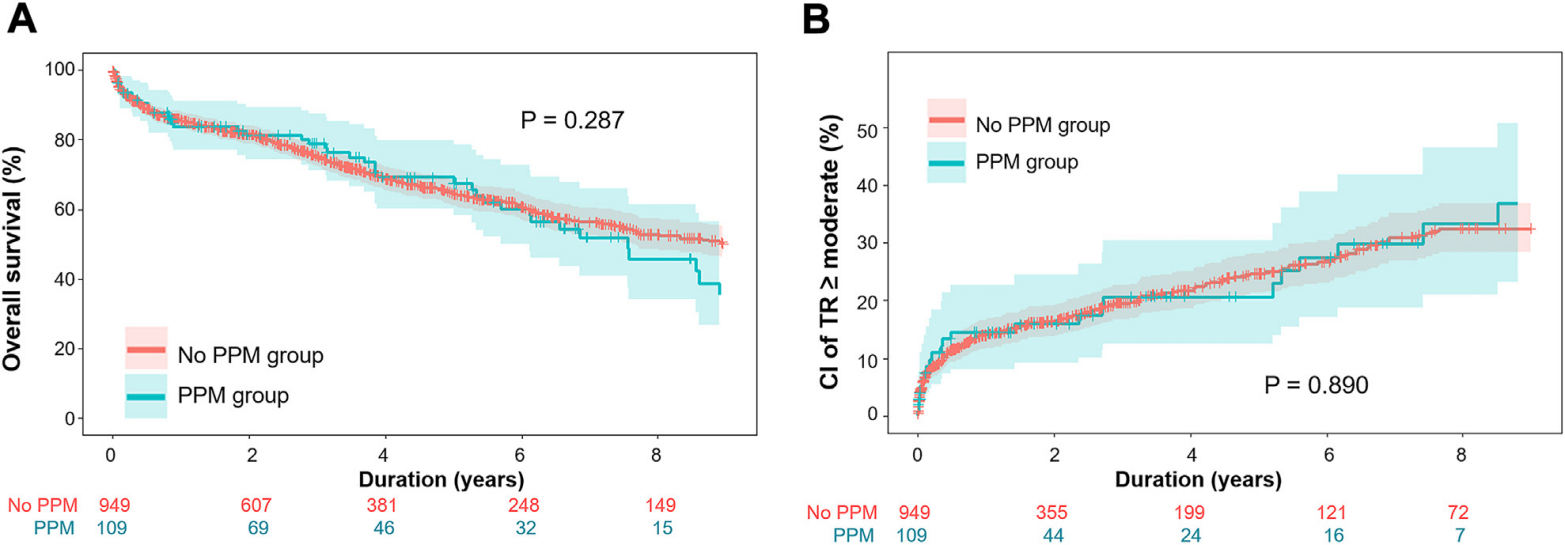
(A) Overall survival and (B) cumulative incidence of recurrence moderate or greater tricuspid regurgitation (TR) in patients with permanent pacemaker (PPM) implantation and without PPM implantation after tricuspid valve repair.

## Comment

This study demonstrates 2 main findings. First, the incidence of PPM implantation after TV repair was relatively high, at 10.3%. Second, the preoperative characteristics and concomitant procedure, such as age, history of mediastinal radiation, concomitant MVR and AVR, and LVAD implantation were associated with the incidence of PPM implantation after TV repair.

The recent Cardiothoracic Network trial for mitral regurgitation has raised the safety issue of tricuspid repair for PPM implantation.^4^ Previous reports have reported the incidence of new pacemaker implantation after tricuspid valve repair to vary from 2.4% to 16%.^3^^,^^4^^,^^6^ In this series, the overall incidence of PPM implantation after TV repair was 10.4%. Although PPM implantation could be affected many factors like patient population, device types, and postoperative strategy related to timing of PPM insertion, these data suggest that the risk of needing a pacemaker at the time of tricuspid repair is real. This relatively high incidence of PPM implantation can be affected by various factors other than atrioventricular nodal injury that may occur during TV repair. In fact, 15.6% of the pacemakers were for ventricular arrhythmias and 19.3% of pacemakers were indicated for junctional or sinus bradycardia more likely related to right atrial manipulation or surgical ablation.

Few series have identified predictors of pacemaker requirement. Some reports suggested that a flexible band can be a protective factor for PPM implantation,^7^^,^^8^ yet there was no significant difference in PPM implantation according to flexible band vs rigid ring in this study. This paper showed several preoperative factors and concomitant procedure were associated with PPM implantation. Preoperative radiation therapy was a powerful risk factor for PPM implantation with an OR of 4.10. Tissue fibrosis induced by mediastinal radiation may affect the sinus node or atrioventricular node and predispose to injury during surgery. Although the mechanisms of this phenomenon cannot be elucidated in this study, intraoperative epicardial pacemaker lead insertion could be considered to further minimize the impact of a pacemaker lead on tricuspid repair durability. The protective effect of concurrent LVAD may in part be related to these patients being less affected by ventricular arrhythmias or bradycardia when the body is being supported by mechanical circulatory assist.

Limitations of this study include being a single institution series, inclusion of a large number of left ventricular assist devices typically found only in academic centers, and extending over a period of 22 years where the technology and indications for pacemaker placement have evolved.

In conclusion, the likelihood of PPM implantation after TV repair is relatively high, but PPM implantation does not affect the early and long-term clinical outcomes. Older age, prior mediastinal radiation, and concomitant MVR and AVR are risk factors, and concomitant LVAD implantation is a protective factor for PPM implantation after TV repair.
