# Supplementary material for: Incidence and Risk Factors for Permanent Pacemaker Implantation After Tricuspid Valve Repair
Source: Ann Thorac Surg Short Rep. 2024 Aug 28;3(1):37–41. doi: 10.1016/j.atssr.2024.08.003 (PMC11910800; doi:10.1016/j.atssr.2024.08.003)
Supplement: Supplementary Table 1-2 [file mmc1.docx]

**Supplemental table 1.** Prosthetic ring types in tricuspid valve.

| Variables | All  (n = 1021) | PPM  (n = 107) | No PPM  (n = 914) |
| --- | --- | --- | --- |
| Tri-Ad | 487 (47.7) | 51 (47.7) | 436 (47.7) |
| Tailor | 372 (36.4) | 38 (35.5) | 334 (36.5) |
| Carpentier | 106 (10.4) | 13 (12.1) | 93 (10.2) |
| Duran AnCore | 55 (5.4) | 5 (4.7) | 50 (5.5) |
| Other ring | 1 (0.1) | 0 (0) | 1 (0.1) |

Values are n (%). PPM=Permanent pacemaker

**Supplemental table 2.** Early and long-term outcomes

| Variables | All  (n = 1058) | PPM  (n = 109) | No PPM  (n = 949) | P value |
| --- | --- | --- | --- | --- |
| Early outcomes |  |  |  |  |
| Death | 65 (6.1) | 6 (5.5) | 59 (6.2) | 0.769 |
| Low cardiac output syndrome | 61 (5.8) | 6 (5.5) | 55 (5.8) | 0.894 |
| Stroke | 35 (3.3) | 5 (4.6) | 30 (3.2) | 0.396 |
| Acute kidney injury | 108 (10.2) | 7 (6.4) | 101 (10.6) | 0.168 |
| Respiratory complication | 143 (13.5) | 14 (12.8) | 129 (13.6) | 0.828 |
| Reoperation for bleeding | 71 (6.7) | 3 (2.8) | 68 (7.2) | 0.081 |
| Mediastinitis | 14 (1.3) | 2 (1.8) | 12 (1.3) | 0.648 |
| Length of stay (day, median) | 19.2 ± 20.1 | 17.6 ± 12.9 | 19.4 ± 20.8 | 0.191 |
| Long-term outcomes |  |  |  |  |
| 5-year survival | 65 (61-68) | 65(61-68) | 67(58-78) | 0.287 |
| 10-year survival | 44 (40-49) | 46(42-52) | 26(15-45) |  |
| 5-year CI moderate TR | 24.2 (21.1-27.6) | 20.7 (12.5–30.3) | 24.7 (21.3–28.2) | 0.890 |
| 10- year CI moderate TR | 33.3 (29.2-37.4) | 36.9 (23.2-50.6) | 32.9 (28.6-37.2) |  |
| 5-year CI severe TR | 8.5 (6.5-10.9) | 7.2(2.9-14.1) | 8.7(6.5-11.3) | 0.621 |
| 10-year CI severe TR | 11.6 (8.9-14.7) | 7.2(2.9-14.1) | 12.1 (9.1-15.4) |  |
| 5-year CI TV reoperation | 1.0 (0.5-1.8) | 2.0 (0.4-6.5) | 0.8 (0.4-1.7) | 0.602 |
| 10-year CI TV reoperation | 2.0 (1.1-3.7) | 2.0 (0.4-6.5) | 2.1 (1.0-3.8) |  |

Values are mean ± standard deviation, n (%) or median (interquartile range). CI = cumulative incidence, PPM=Permanent pacemaker, TR=tricuspid regurgitation, TV=tricuspid valve,
